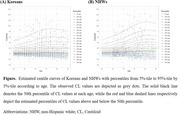# Age‐related amyloid‐β dynamics modeled with the generalized additive model for location, scale, and shape (GAMLSS) across diverse populations: cross‐sectional trajectories and longitudinal validation

**DOI:** 10.1002/alz70856_104711

**Published:** 2026-01-07

**Authors:** Min Young Chun, Sungjoo Lee, Michael S. W. Weiner, Suzanne E. Schindler, Daeun Shin, Heekyung Kang, Sohyun YIM, Eun Hye Lee, Kyunga Kim, Hee Jin Kim, Duk L. Na, Jun Pyo Kim, Sang Won Seo

**Affiliations:** ^1^ Samsung Medical Center, Sungkyunkwan University School of Medicine, Seoul, Korea, Republic of (South); ^2^ Yonsei University College of Medicine, Seoul, Korea, Republic of (South); ^3^ Yongin Severance Hospital, Yonsei University Health System, Yongin, Gyeonggi‐do, Korea, Republic of (South); ^4^ Samsung Medical Center, Gangnam‐gu, Seoul, Korea, Republic of (South); ^5^ University of California, San Francisco, San Francisco, CA, USA; ^6^ Washington University School of Medicine, St. Louis, MO, USA; ^7^ Samsung Medical Center, Gangnam‐gu, Seoul, Korea, Republic of (South); ^8^ Indiana Alzheimer Disease Research Center, Indiana University School of Medicine, Indianapolis, IN, USA; ^9^ Research Institute for Future Medicine, Samsung Medical Center, Gangnam‐gu, Seoul, Korea, Republic of (South)

## Abstract

**Background:**

Alzheimer's disease is characterized by early amyloid‐β (Aβ) accumulation, even in cognitively unimpaired (CU) individuals, yet age‐related Aβ trajectories remain poorly understood. We developed and validated age‐related Aβ positron emission tomography ( trajectories using a statistical model in CU individuals, and examined the effects of sex and *APOE* ε4.

**Method:**

We analyzed 849 CU Korean participants and 521 CU non‐Hispanic Whites (NHWs) after propensity score matching. Age‐related Aβ PET trajectories were modeled using the generalized additive model for location, scale, and shape (GAMLSS) based on cross‐sectional data and validated with longitudinal data. Subgroup analyses explored the influence of *APOE* ε4 and sex on age‐related Aβ PET trajectories.

**Result:**

The age‐related centile curves of Aβ PET centiloid values showed stable distributions in the lower percentiles. Aβ values increased with age in the upper percentiles for both Koreans and NHWs. NHWs demonstrated steeper trajectories of Aβ accumulation, particularly among *APOE* ε4 carriers, compared to Koreans. The reliability of this cross‐sectional method was confirmed through robust calibration using the study's own longitudinal dataset.

**Conclusion:**

Our study developed a statistical model of age‐related Aβ PET trajectories using cross‐sectional data, validated with longitudinal data. NHWs had steeper trajectories of AB accumulation compared to Koreans. The results suggest differences in population‐specific patterns of Aβ accumulation.